# Isolation and characterization of mutated alcohol oxidases from the yeast *Hansenula polymorpha *with decreased affinity toward substrates and their use as selective elements of an amperometric biosensor

**DOI:** 10.1186/1472-6750-7-33

**Published:** 2007-06-13

**Authors:** Kostyantyn V Dmytruk, Oleh V Smutok, Olena B Ryabova, Galyna Z Gayda, Volodymyr A Sibirny, Wolfgang Schuhmann, Mykhailo V Gonchar, Andriy A Sibirny

**Affiliations:** 1 Institute of Cell Biology, NAS of Ukraine, Drahomanov Street 14/16, Lviv 79005, Ukraine; 2 Department of Metabolic Engineering, Rzeszow University, Cwiklinskiej 2, 35-601 Rzeszow, Poland; 3 Anal. Chem.-Elektroanalytik and Sensorik, Ruhr-Universität Bochum, Universitätsstr. 150, D-44780 Bochum, Germany

## Abstract

**Background:**

Accurate, rapid, and economic on-line analysis of ethanol is very desirable. However, available biosensors achieve saturation at very low ethanol concentrations and thus demand the time and labour consuming procedure of sample dilution.

**Results:**

*Hansenula polymorpha *(*Pichia angusta*) mutant strains resistant to allyl alcohol in methanol medium were selected. Such strains possessed decreased affinity of alcohol oxidase (AOX) towards methanol: the K_M _values for AOX of wild type and mutant strains CA2 and CA4 are shown to be 0.62, 2.48 and 1.10 mM, respectively, whereas V_max _values are increased or remain unaffected. The mutant AOX alleles from *H. polymorpha *mutants CA2 and CA4 were isolated and sequenced. Several point mutations in the AOX gene, mostly different between the two mutant alleles, have been identified. Mutant AOX forms were isolated and purified, and some of their biochemical properties were studied. An amperometric biosensor based on the mutated form of AOX from the strain CA2 was constructed and revealed an extended linear response to the target analytes, ethanol and formaldehyde, as compared to the sensor based on the native AOX.

**Conclusion:**

The described selection methodology opens up the possibility of isolating modified forms of AOX with further decreased affinity toward substrates without reduction of the maximal velocity of reaction. It can help in creation of improved ethanol biosensors with a prolonged linear response towards ethanol in real samples of wines, beers or fermentation liquids.

## Background

The detection and quantification of alcohols with high selectivity and accuracy is required in many different areas. Accurate and rapid measurement of ethanol is important in clinical and forensic practice to analyse human body fluids, e.g. blood, serum, saliva, urine, breath and sweat, among others. The food, beverage (wine, beer and spirits), and pulp industries also require simple, correct, fast, and economic analytical methods to control fermentation processes and the quality of products.

Enzymatic and biosensor approaches are among the most convenient analytical methods for this purpose. Yeast alcohol oxidase (AOX; alcohol:O_2 _oxidoreductase, EC 1.1.3.13) has been extensively used for the determination of lower primary alcohols and formaldehyde [1-6]. Significant progress in selection of optimal transducers, immobilization and stabilization of enzyme has been achieved. However, the affinity of AOX to ethanol is rather high, and direct AOX-based measurements of a target analyte, *e.g*. ethanol in real samples of wines, beers or fermentation cultures, is complicated and economically detrimental due to the necessity of diluting the samples. This is especially important in automatic electrochemical devises such as biosensors since the additional step of sample dilution causes significantly increasing costs of analysis. In order to overcome this inconvenience, obtaining modified forms of AOX with decreased affinity towards substrates and a wide range of linear response is highly desirable.

In this work, we report on the selection of methylotrophic yeast *Hansenula polymorpha *mutants which produce modified AOXs with decreased affinities toward substrates and with maximal velocities that increased or remained unaffected. We communicate on the identification of substitutions in mutant alleles, purification of mutant AOXs, and study of applicability of the modified enzyme in amperometric biosensor development.

## Results and discussion

### The generation, characterization, sequencing, and purification of the mutant forms of AOX

For isolation of the mutant forms of AOX (mAOX) with decreased affinity to substrates, a positive selection procedure was developed. Allyl alcohol was used as the selective agent in the medium with methanol as sole carbon and energy source. The rationale for the developed selection scheme was as follows. Allyl alcohol is oxidized by AOX with the formation of highly toxic acrolein. In the medium with the mixture of methanol (0.5%) and allyl alcohol (minimal inhibitory concentration was found to be 0.3 mM, see Materials and Methods section), mutants defective in AOX could not arise. Mutants producing lower amounts of acrolein and enough formaldehyde for growth could be selected. One of the reasons for such an event would be the decrease of affinity of AOX toward substrates while retaining high enough reaction velocity. This selection scheme would produce few or no mutants with decreased rate of AOX reaction as they would not grow efficiently due to the negligible amount of formaldehyde produced. Altogether, 125 mutants of *H. polymorpha *strain DL-1-356 that were able to growth on allyl alcohol containing medium were chosen for further selection. Mutants with decreased enzyme affinity towards ethanol were screened by a plate colony assay as described in the Material and Methods section. Finally, two positive colonies (strains CA2 and CA4) which stained in the presence of 15 mM ethanol, but not 5 mM or 10 mM, were selected.

The kinetic parameters of AOX from the selected mutant strains were evaluated. K_M _values towards methanol for the parental strain and the CA2 and CA4 mutants were calculated as 0.62, 2.48, and 1.10 mM, respectively (Table 1). At the same time, V_max _of AOX from the fore mentioned strains was 27.4, 66.7 and 31.3 μmol of atomic O·min^-1^·mg^-1 ^of protein at 20°C, respectively (Table 1). Thus, allyl alcohol/methanol-based selection is suitable for obtaining *H. polymorpha *mutants with decreased AOX substrate affinity and without decreased maximal velocity of the specific enzyme reaction.

**Table 1:** K_M _and V_max _values of AOX towards methanol for natural and mutant *H. polymorpha *strains.

*H. polymorpha *strain	К_М_, mM	V_max_, μmol of atomic O·min^-1^·mg^-1 ^of protein at 20°C	Sigma factor, σ	Linear regression coefficient R for reciprocal plot
DL-1-356	0.62	27.4	0.084	0.992
CA2	2.48	66.7	0.040	0.958*
CA4	1.10	31.3	0.130	0.990

* A relatively low value of linear regression coefficient R for reciprocal plot for CA2-AOX can be explained by decreased activity measured and with accordingly increased error value

**Figure 1:**
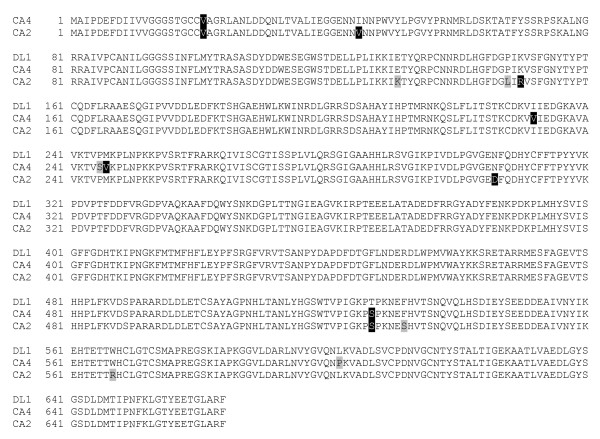
Alignment of deduced amino acid sequences for AOX from the mutant strains of *H. polymorpha *CA2, CA4 and the wild type DL1.

Mutant alleles of AOX gene from CA2 and CA4 strains were cloned and sequenced. Nucleotide sequences of the mutant alleles were translated into amino acid sequences and compared with that of the wild-type strain (Figure 1). Several amino acid substitutions were identified which apparently cause decreased affinity towards ethanol. It was found that each mutant allele contains numerous substitutions (9 for AOX allele from CA2 and 6 for allele from CA4; of them only 2 substitutions are identical). One can note that the number of point mutations within the mutant alleles is rather high. However, it should be emphasized that the colonies of allyl-alcohol resistant mutants were stored on a selective medium for several weeks up to month. This allowed for the gradual accumulation of several favourable mutations for growth of mutants on allyl alcohol/methanol containing medium. In the other words, natural selection in allyl alcohol/methanol resistant mutants occurred by the accumulation of successive spontaneous or acrolein induced mutations.

Both strains are shown to have I > V substitutions in position 21 and T > S in position 527. In addition, the AOX sequence of CA2 strain has amino acid substitutions I > V (45), E > K (131), P > L (148), K > R (150), N > D (306), F > S (532), and W > R (567). The mutant protein in strain CA4 has a lower substitution number which includes: I > V (232), P > S (245), M > V (246), and L > P (602). The level of the substrate affinity reduction correlates with the average quantity of amino acid substitutions.

The AOXs were purified. Samples of CA2 and CA4 mAOXs as well as native AOX (nAOX) were obtained with quantities close to 100 mg with specific activities of 16, 23 and 19 μmol·min^-1^·mg^-1^, respectively. The average yield of chromatographically pure AOX samples was close to 20% of the initial amount (Table 2). Homogeneity of the resulting proteins was confirmed by electrophoresis. The molecular weights of mutant proteins, according to SDS-PAGE electrophoresis data, are close to that of the natural protein [7]. Purified enzymes were used for construction of an amperometric biosensor.

**Table 2:** Preparative isolation and purification of AOX from the mutant strains (on the example of CA2).

Stage	Result	Specific AOX activity, μmol·min^-1^·mg^-1 ^of protein (U·mg^-1^)	Total AOX yield, U (%)*
Cell growth	17.7 g of cells	0.35 (per 1 mg of cells)	6130 (100%)
Cell disintegration	Crude extract, mg 2300	2.0	4600 (75%)
Fractionation by ammonium sulphate (30 – 70%)	Protein precipitate (70% of ammonium sulphate saturation), mg 750	4.0	3000 (49%)
Chromatography on DEAE – Toyopearl 650 M	Protein sample, mg 75	16.0	1200 (20%)

* 1 Unit is equal 1 μmol of converted substrate per 1 min at standard conditions of assay (20°C).

### Bioanalytic characterization of an amperometric biosensor based on a mutant form of AOX

**Figure 2:**
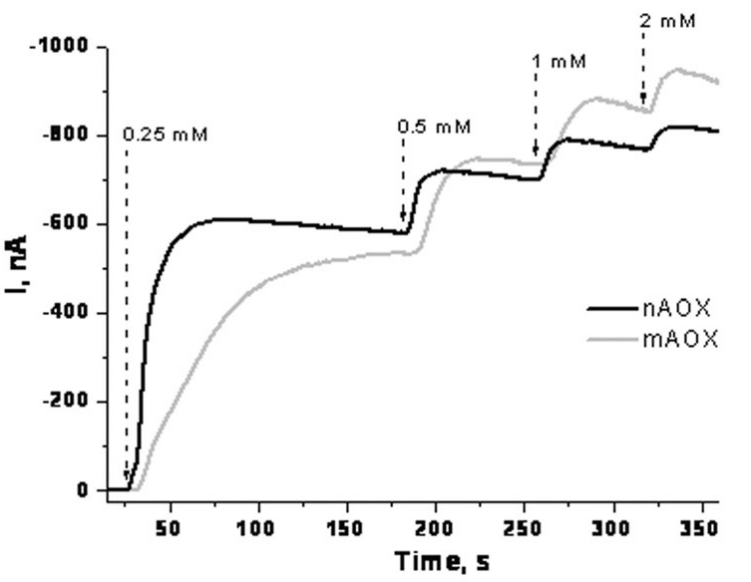
Chronoamperometric assay of ethanol by HRP/Os-Ap59//AOX/CP9 biosensors based on the native (nAOX) and mutated AOX (mAOX) forms of alcohol oxidase and the derived calibration curves. Arrays indicate step-wise addition of ethanol in different concentrations.

An amperometric biosensor based on mAOX (from the CA2 mutant) and peroxidase (HRP), architected as *HRP/Os-AP59//AOX(CA2)/CP59*, was constructed according to [8]. Characterisation of the enzymatic properties of mAOX was performed using nAOX as a reference. The typical dynamic ranges for AOX-modified sensors to ethanol are presented in Figure 2.

**Figure 3:**
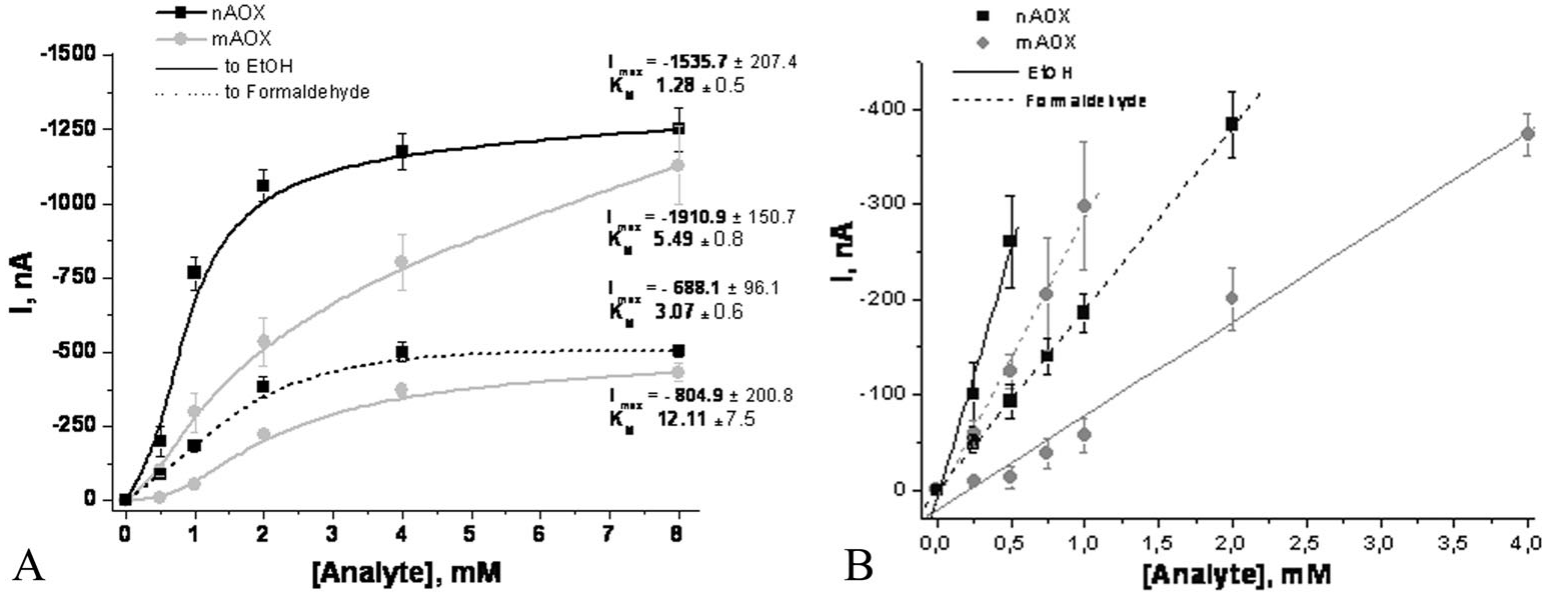
Calibration curves and calculated K_M _values of sensors based on the different forms of AOX toward ethanol (EtOH) and formaldehyde **(A) **and dynamic linear ranges of the corresponded sensors toward both analytes **(B)**.

In addition to alcohols (e.g. ethanol or methanol), formaldehyde is also a substrate for AOX [9]. The calibration curves of the biosensors response to ethanol and formaldehyde clearly show the lower affinities toward both substrates of mAOX compared to wild-type enzyme (Figure 3A).

As was mentioned before, the natural and mutant forms of AOX were shown to differ in K_M _value towards methanol in solution: the mutant enzyme possesses 4-fold increased K_M _value and, thus, decreased substrate affinity (Table 1). The results obtained by use of the AOX-immobilised amperometric sensor show good agreement with those obtained with AOXs in solution. Thus, the K_M _value of a nAOX-immobilised electrode were 1.28 mM towards ethanol and 3.07 mM towards formaldehyde, whereas these values for the mAOX-immobilised electrode were increased to 5.40 mM towards ethanol and 12.1 mM towards formaldehyde. These results also show that mutant AOXs have a decreased affinity not only to the physiological substrate methanol, but also to other substrates, such as ethanol and formaldehyde. It is interesting to note that the I_max _values for ethanol and formaldehyde of nAOX/mAOX-immobilised electrodes were 1535.7/1910.9 nA and 688.1/804.9 nA, respectively. The I_max _values of the constructed sensors did not differ significantly (Figure 3A). Otherwise the linear dynamic range of the mAOX-immobilised electrode to ethanol arranged 4 mM vs 0.5 mM for nAOX-immobilised electrodes (Figure 3B).

**Figure 4:**
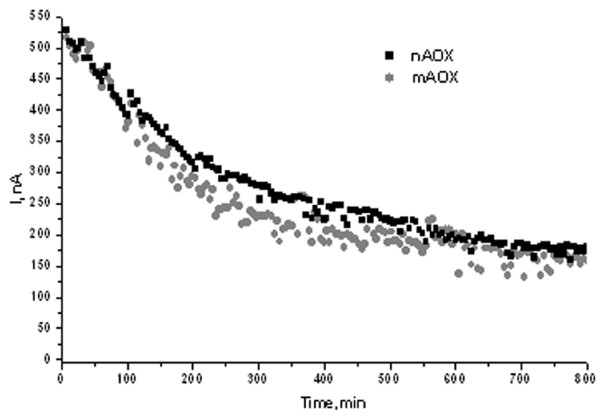
Comparison of an operational stability of nAOX and mAOX-modified electrodes.

The study of operational stability and inactivation kinetics of the constructed biosensors revealed that the sensors based on either mutant or natural enzymes are very similar (Figure 4). Both sensors retained 50% of their activity after 5 h of continuous exploitation. The storage stability for both constructed biosensors was better than 16 days with 50% current drop after 14 days of storage (data not shown).

Thus, the constructed amperometric biosensor based on mAOX (CA2) was characterised by decreased affinity towards analyzed substrates without altered operational stability of the sensor.

## Conclusion

In this study, we describe a novel selection procedure for isolation of the mutant forms of AOX, and their use as a suitable bioelement for biosensor technologies. The created biosensor based on mAOX (from the strain CA2) was characterised by a decreased affinity towards analyzed substrates and slightly increased V_max_. Furthermore, the operational stability of the mAOX-immobilised electrode was not affected and remained similar to an electrode based on the natural enzyme. The described methodology opens up the possibility for construction of biosensors appropriate for precise, rapid, and cheap analysis of target analytes, *e.g*. ethanol in real samples of wines, beers or fermentation cultures. For further improvement of mAOX as a biosensor element, various strategies could be used: 1) direct design of mutant forms of AOX based on results obtained from site-specific mutagenesis; 2) evolutionary engineering of mutant forms of AOX through continuous cultivation of the corresponding strains in medium with increased concentrations of allyl alcohol; 3) determination of crystal structures of an AOX of *H. polymorpha*, elucidation of the structural bases involved in the enzyme-substrate interaction and subsequent direct modification of enzyme characteristics.

## Methods

### Strain and media

*Escherichia coli *strain DH5α (Φ80d*lacZ*ΔM15, *recA*1, *endA*1, *gyrA*96, *thi*-1, *hsdR*17(r_K_^-^, m_K_^+^), *supE*44, *relA*1, *deoR*, Δ(*lacZYA-argF*)U169) was used in experiments which required a bacterial host. DH5α was grown at 37°C in rich (LB) medium as described in Sambrook et al. [10]. Transformed *E. coli *cells were maintained on a medium containing 100 mg·L^-1 ^of ampicillin.

*H. polymorpha *DL-1-356 (*leu2*) [11] derived from DL1 (ATCC 26012) strain was grown on YPD or synthetic Burkholder medium [12] supplemented with various carbon sources: glucose, methanol and glycerol. Concentration of carbon sources was 1% (w/v or v/v) unless stated otherwise. Leucine was added to final concentration of 40 mg·L^-1 ^if required. For solid media, agar was added to 1.5% (w/v). Yeast cells were grown in Erlenmeyer flasks with continuous shaking (200 rpm) at 37°C.

### Mutant isolation

For mutant isolation, cells of the strain *H. polymorpha *DL-1-356 were cultivated in liquid medium with glucose until midexponential growth phase, washed with distilled water, diluted to 10^6 ^cell·mL^-1 ^and irradiated with a dose of UV light that allows 10% cell survival. Then, cells were diluted and plated on the solid Burkholder medium containing methanol (0.5%) and allyl alcohol (0.3 mM). Allyl alcohol was applied as a selective agent; it is oxidized by AOX to acrolein. This compound is toxic for yeast and results in the death of cells with a high AOX activity. On the other hand, AOX activity is essential for growth in medium with methanol as a sole carbon source. Thus, the medium with a mixture of methanol and allyl alcohol will provide a growth of the cells with decreased AOX activity and/or decreased AOX affinity to alcohols. Mutant colonies grown after 21–25 days of incubation were replica-plated into the same medium for confirmatory selection. Mutants with decreased enzyme affinity towards ethanol were screened by a plate colony assay, on the basis of visualization of AOX activity by the rate of hydrogen peroxide formation in reaction with ethanol as monitored by the peroxidative oxidation of *o*-dianisidine in the presence of peroxidase resulted purple colour of colonies [9]. For this purpose, the plates were incubated at 37°C for 2 days and the colonies formed were replica plated onto agar plates supplemented with glycerol. After 18 h of incubation at 37°C, the plates were overlaid with 9 ml of the alcohol oxidase activity assay mixture, containing 50 mM potassium phosphate buffer (pH 7.5), 0.7% (wt/vol) agar, digitonin (1 mg·mL^-1^), *o*-dianisidine (0.5 mg·mL^-1^), peroxidase Sigma RZ 1.1 (0.13 mg·mL^-1^), and different (0–15 mM) concentrations of ethanol. The overlying assay mixture was allowed to set, and the plates were incubated at 37°C for up to 1 h. Colonies that stained purple only in the presence of 15 mM ethanol, but not 5 mM or 10 mM, were selected.

### Evaluation of kinetic parameters of mutant AOX

AOX affinity towards methanol was evaluated via a kinetic study of enzyme activity in crude cell-free extracts of parental [13] and mutant *H. polymorpha *strains CA2 and CA4 in the presence of different methanol concentrations (0–100 mM) in the peroxidase reaction with ABTS (2,2'-azino-bis(3-ethylbenzothiazoline-6-sulphonic acid). K_M _was calculated from the initial reaction rates, depending on substrate concentration, via the equation:

V=Vmax⁡[S]KM+[S],
 MathType@MTEF@5@5@+=feaafiart1ev1aaatCvAUfKttLearuWrP9MDH5MBPbIqV92AaeXatLxBI9gBaebbnrfifHhDYfgasaacH8akY=wiFfYdH8Gipec8Eeeu0xXdbba9frFj0=OqFfea0dXdd9vqai=hGuQ8kuc9pgc9s8qqaq=dirpe0xb9q8qiLsFr0=vr0=vr0dc8meaabaqaciaacaGaaeqabaqabeGadaaakeaacqWGwbGvcqGH9aqpdaWcaaqaaiabdAfawnaaBaaaleaacyGGTbqBcqGGHbqycqGG4baEaeqaaOGaei4waSLaem4uamLaeiyxa0fabaGaem4saS0aaSbaaSqaaiabd2eanbqabaGccqGHRaWkcqGGBbWwcqWGtbWucqGGDbqxaaGaeiilaWcaaa@4020@

where V – reaction rate, S – substrate concentration, V_max _and K_M _– kinetic constants. Constants were calculated with a double-reciprocal method of Lineweaver-Burk [14] using Excel (MS Office pack).

### Isolation and analyses of nucleic acids

The 1995 bp coding regions for the AOX was amplified by PCR using the following primers LV1 (5'-CCC AAG CTT ATG GCC ATT CCT GAC GAA TTC-3'); LV2 (5'-CCC AAG CTT TTA GAA TCT GGC AAG TCC G-3') and DNA was extracted from the *H. polymorpha *DL-1-356, and mutant strains CA2 and CA4. The primers were designed based on the AOX sequence of the *H. polymorpha *CBS 4732 [15]. The amplified AOX alleles and the vector pUC57, were cut with *Hind*III and ligated. The resulting plasmids were sequenced. Sequencing of these DNA fragments was performed on both strands. Alignments of putative amino acid sequences were carried out by the programs MultAlin [16] and BOXSHADE 3.21 [17]. The nucleotide sequences of *H. polymorpha *AOX wild type (DL-1-356), mutant alleles CA2 and CA4 were deposited in GenBank and were assigned the accession nos AM690088, AM690089 and AM690090, respectively.

### Molecular biology techniques

Preparations of total DNA from yeast were carried out by using the DNeasy^® ^Tissue Kit (Qiagen, Germany). Plasmid DNA isolations from *E. coli *were carried out by using NucleoSpin^® ^Plasmid (Macherey-Nagel, Germany).

### AOX purification

Highly purified AOX was isolated from a cell-free extract of the isolated mutants, as described elsewhere [8,18], by means of a two-step ammonium sulphate fractionation (at 30 and 70% of saturation) followed by dialysis and ion exchange chromatography on DEAE-Toyopearl 650 M.

### Construction of amperometric biosensor

Amperometric biosensors based on mAOX (CA2) were evaluated using constant-potential amperometry in a three-electrode configuration with a Ag/AgCl/KCl (3 M) reference electrode and a Pt-wire counter electrode. The applied working potential was -50 mV vs Ag/AgCl. Amperometric measurements were carried out using a bipotentiostat (EP 30, Biometra, Göttingen, Germany) connected to a personal computer via a RS232 port for data acquisition. Between experiments, the enzyme electrodes were stored in 20 mM phosphate buffer, pH 7.2, at 4°C.

Bioselective layer of sensor was constructed according to [8]. The structure of the bi-enzyme electrode was configured as *HRP/Os-Ap59//AOX(CA2)/CP9 *and included two layers: the inner, with horse radish peroxidase (*HRP*) electrochemically precipitated in the presence of carboxylate-containing polymer modified with an osmium-pyridyl complex (*Os-Ap59*), and the outer, with mAOX (CA2) immobilized via cathodic precipitation in the presence of an amino-containing polymer (*CP9*). The Os-containing polymer served as a redox-mediator in the electrochemical reaction. Platinized graphite rods (type RW001, 3.05 mm diameter, Ringsdorff Werke, Bonn, Germany) were used as working electrodes which were sealed in a glass tube using epoxy thus forming disk electrodes.

### Determination of biosensors operational stability

The determination of operational stability and inactivation kinetics of the constructed biosensors was performed by using the analyzing system "Olga" (output of sensors to 1 mM ethanol, ejection time 2 min) [19].

## Authors' contributions

KVD carried out the molecular genetic studies, participated in the sequence alignment and drafted the manuscript. OVS constructed and characterised the amperometric biosensors. OBR performed mutant isolation and evaluation of kinetic parameters of the AOX mutants. GZG purified enzymes. OVS, OBR and MVG co-drafted the manuscript. VAS, WS and MVG supervised some of the work and participated in the design of the experiments. MVG, WS and AAS provided guidance and suggestions for experimental design, analyzed data, and edited the manuscript. VAS and AAS conceived the presented study. AAS is the author of the method for selection of mutants with affected affinity of alcohol oxidase toward substrates. All authors read and approved the final version of the manuscript.
